# Near-Infrared Fluorescent Imaging for Monitoring of Treatment Response in Endometrial Carcinoma Patient-Derived Xenograft Models

**DOI:** 10.3390/cancers12020370

**Published:** 2020-02-06

**Authors:** Tina Fonnes, Elin Strand, Kristine E. Fasmer, Hege F. Berg, Heidi Espedal, Kristina Sortland, Ingunn Stefansson, Line Bjørge, Ingfrid S. Haldorsen, Camilla Krakstad, Emmet McCormack

**Affiliations:** 1 Centre for Cancer Biomarkers CCBIO, Department of Clinical Science, University of Bergen, 5021 Bergen, Norway; tina.fonnes@uib.no (T.F.); Elin.Strand@helse-bergen.no (E.S.); hege.berg@uib.no (H.F.B.); Line.Bjorge@uib.no (L.B.); camilla.krakstad@uib.no (C.K.); 2 Department of Obstetrics and Gynecology, Haukeland University Hospital, 5021 Bergen, Norway; 3 Center for Nuclear Medicine/PET, Department of Radiology, Haukeland University Hospital, 5021 Bergen, Norway; kristine.eldevik.fasmer@helse-bergen.no; 4 Department of Biomedicine, University of Bergen, 5021 Bergen, Norway; heidi.espedal@uib.no (H.E.); kristinasortland@hotmail.com (K.S.); 5 Department of Pathology, Haukeland University Hospital, 5021 Bergen, Norway; ingunn.stefansson@uib.no; 6 Mohn Medical Imaging and Visualization Centre, Department of Radiology, Haukeland University Hospital, 5021 Bergen, Norway; ingfrid.helene.salvesen.haldorsen@helse-bergen.no; 7 Section for Radiology, Department of Clinical Medicine, University of Bergen, 5021 Bergen, Norway

**Keywords:** endometrial carcinoma, epithelial cell adhesion molecule, near-infrared fluorescent imaging, patient-derived xenografts, orthotopic mouse model

## Abstract

Imaging of clinically relevant preclinical animal models is critical to the development of personalized therapeutic strategies for endometrial carcinoma. Although orthotopic patient-derived xenografts (PDXs) reflecting heterogeneous molecular subtypes are considered the most relevant preclinical models, their use in therapeutic development is limited by the lack of appropriate imaging modalities. Here, we describe molecular imaging of a near-infrared fluorescently labeled monoclonal antibody targeting epithelial cell adhesion molecule (EpCAM) as an in vivo imaging modality for visualization of orthotopic endometrial carcinoma PDX. Application of this near-infrared probe (EpCAM-AF680) enabled both spatio-temporal visualization of development and longitudinal therapy monitoring of orthotopic PDX. Notably, EpCAM-AF680 facilitated imaging of multiple PDX models representing different subtypes of the disease. Thus, the combined implementation of EpCAM-AF680 and orthotopic PDX models creates a state-of-the-art preclinical platform for identification and validation of new targeted therapies and corresponding response predicting markers for endometrial carcinoma.

## 1. Introduction

Endometrial carcinoma is a malignancy originating in the uterine mucosa, and is currently the 6th most common cancer in females worldwide [1]. Estimates suggest that there were close to 400,000 new cases of endometrial carcinoma resulting in approximately 90,000 deaths globally in 2018 [1]. Hysterectomy with bilateral salpingo-oophorectomy, with or without lymphadenectomy, is the primary treatment for endometrial carcinoma. In total, 15–20% of patients will experience recurrence [2,3], which carries a poor prognosis (3 year survival of 8% and 14% for pelvic and distant recurrence, respectively [4]). These patients, and patients that present with advanced disease at time of diagnosis, usually receive adjuvant chemotherapy. However, response rates are modest with limited survival benefit [5]. With rising incidence of endometrial carcinoma in North America and Europe (up to 19 cases per 100,000), there is a critical unmet need to develop novel therapeutics for the anticipated increase in number of recurrent patients [6]. 

There are currently few targeted therapies available for the treatment of recurrent endometrial cancer patients, e.g., pembrolizumab for endometrial tumors with a high degree of microsatellite instability [7,8]. It is clear that further delineation of key molecular characteristics including histologic type (low and high grade endometrioid or non-endometrioid tumors), biomarker expression (i.e., estrogen and progesterone receptor status), and genomic classification (polymerase ε ultramutated (POLE), microsatellite instability hypermutated, copy number high and copy number low tumors [9]) will be critical to improve the probability of beneficial outcomes in the personalized care of these patients [2]. Thus, the development and application of clinically relevant models of endometrial carcinoma, which accurately reflect these key clinical characteristics, will be crucial to translational development of personalized therapies. Until recently, clinically relevant preclinical models have been lacking for endometrial carcinoma, thus limiting the relevance of translational studies. While both human cell line derived xenografts, and genetically engineered models (GEM) have been employed to study response to several new drugs in vivo [10,11,12,13,14], they fail to reproduce the complexity, heterogeneity and growth rates of primary clinical tumors [15]. In contrast, patient-derived xenograft (PDX) models maintain the tissue architecture, cellular composition and molecular characteristics of tumors from their respective donor patients [15]. Thus, PDX models are not only anticipated to capture both the intra- and inter-tumor heterogeneity of endometrial carcinoma, as has been previously demonstrated for breast, lung and pancreatic cancer, but also to faithfully reproduce clinical responses to chemotherapy [16,17]. Critically, orthotopic PDX models, i.e., where primary biopsies are implanted into the organ/microenvironment of origin, are proposed as a superior paradigm of clinical disease compared with subcutaneous PDX [18,19,20]. However, a major caveat of the orthotopic approach, particularly with patient-derived material, is longitudinal and spatio-temporal monitoring of not only disease development and characterization but also for further intervention studies.

Despite large variations in clinical practice between hospitals, in vivo imaging (including modalities such as transvaginal ultrasound, magnetic resonance imaging (MRI) and computed tomography (CT) with or without positron emission tomography (PET)) play a vital role in both the diagnosis and preoperative risk stratification of endometrial carcinoma patients [21]. Similarly, optical imaging, typically employing bioluminescent reporter genes, have been commonly applied for sensitive, high-throughput imaging of cell line-based xenografts [18,22]. While the use of bioluminescent imaging (BLI) in the development and application of uterine PDXs appears attractive, particularly in comparison to expensive and time-consuming MRI and PET/CT [18], there are caveats. Transduction of bioluminescent reporter genes into primary patient material carries the risk of disrupting the genomic landscape of the selected material. Additionally, each patient sample must be transduced individually, increasing time to use and costs substantially. Ideally, application of optical imaging would utilize a universal imaging contrast reagent, which would permit universal imaging of all PDX.

In vivo near-infrared fluorescent (NIRF) optical imaging using exogenous fluorescent antibodies targeting specific molecular markers has been applied to visualize tumor growth and therapeutic interventions in multiple cancer models [23,24,25]. In the current study we mined for a marker that is universally expressed in endometrial carcinoma, to serve as an imaging target for NIRF imaging of orthotopic PDX models. Subsequently, epithelial cell adhesion molecule (EpCAM) was found to be expressed in 98% of primary uterine patient samples and cell lines. Conjugation of EpCAM to the near-infrared fluorophore Alexa680 yielded EpCAM-AF680, and its application as an optical imaging contrast reagent was found to faithfully depict uterine tumors and metastases in both cell line derived and PDX mouse models. EpCAM-AF680 NIRF imaging was also successfully applied to monitor orthotopic PDX in a therapeutic setting.

## 2. Results

### 2.1. Endometrial Carcinoma Cell Lines Express EpCAM In Vitro

**Figure 1:**
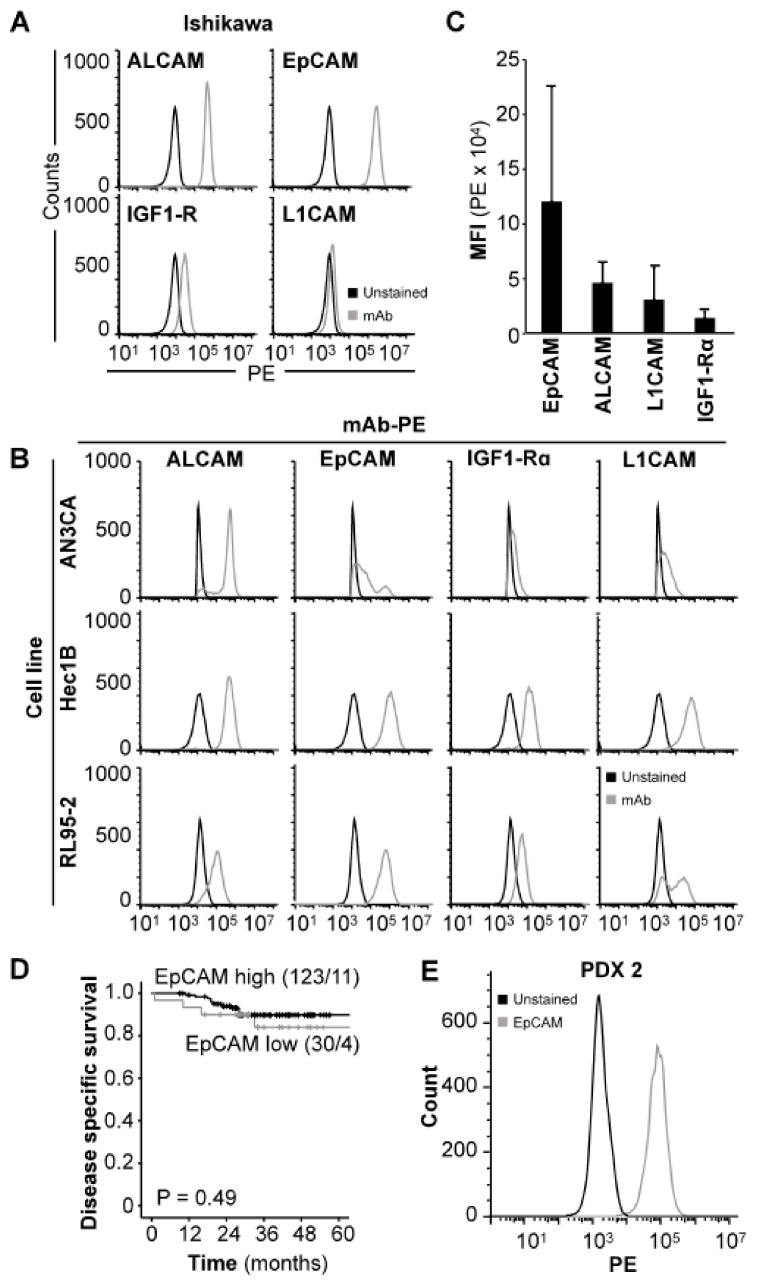
EpCAM is expressed in both endometrial carcinoma cell lines and tumors. Protein expression of ALCAM, EpCAM, IGF-1Rα and L1CAM in Ishikawa (**A**) and AN3CA, Hec1B and RL95-2 (**B**) cell lines demonstrated by flow cytometry using PE-conjugated antibodies. MFI values of EpCAM, ALCAM, IGF-1Rα and L1CAM in the explored cell lines combined (**C**). Kaplan Meier plot illustrating the lack of significant differences in disease specific survival between patients with high or low EpCAM expression in primary tumor (**D**). In vitro protein expression of EpCAM in PDX-derived tumor cells (PDX 2) demonstrated by flow cytometry (**E**). Abbreviations: Activated leukocyte cell adhesion molecule (ALCAM), Epithelial cell adhesion molecule (EpCAM), Insulin-like growth factor 1 receptor alpha (IGF1Rα), L1 cell adhesion molecule (L1CAM), Monoclonal antibody (mAb), Patient-derived xenograft (PDX), and Phycoerythrin (PE).

In order to identify an optimal target for NIRF imaging of xenograft models, selected cell surface proteins (EpCAM, activated leukocyte cell adhesion molecule (ALCAM), insulin-like growth factor 1 receptor alpha (IGF1R), and L1 cell adhesion molecule (L1CAM)) reported to be expressed in endometrial carcinoma [26,27,28,29,30,31,32,33] were screened by flow cytometry in the endometrial carcinoma cell line Ishikawa. EpCAM was found to have the highest expression, with positive staining in 99.9% of cells and a mean fluorescent intensity (MFI) fold increase of 305.3 in comparison to unstained cells (Figure 1A, Table S1). Further evaluation of the same panel in the endometrial carcinoma cell lines AN3CA, Hec1B and RL95-2 (Figure 1B) was performed in order to verify that the high EpCAM expression was not a unique feature of Ishikawa cells. Results demonstrated that all cell lines analyzed presented with positive populations to all antigens in the following order; EpCAM (range: 48.5–99.9%), ALCAM (range: 31.2–99.9%), L1CAM (range: 0.7–96.2%) and IGF1Rα (range: 2.5–66.7%) (Table S1). Additionally, the average MFI of EpCAM (1.2 × 10^5^ +/− 1.0 × 10^5^ a.u.) across the four cell lines was higher compared to ALCAM (4.6 × 10^4^ +/− 1.8 × 10^4 ^a.u.), L1CAM (3.1 × 10^4^ +/− 2.9 × 10^4^ a.u.) and IGF1Rα (1.4 × 10^4^ +/− 5.8 × 10^3^ a.u.) (Figure 1C). The average MFI fold increase in stained relative to unstained cells was also higher for EpCAM (116.4 +/− 128.0) than for ALCAM (44.0 +/− 23.5), L1CAM (23.2 +/− 19.0) and IGF1Rα (11.2 +/− 1.2) (Table S1). Overall, EpCAM was found to be the superior target for antibody-based applications and was selected for further studies.

### 2.2. EpCAM is Highly Expressed in the Majority of Endometrial Carcinoma Primary Tumors

In order to confirm that EpCAM was not just aberrantly expressed on cell line cultures and was in fact ubiquitously expressed in primary tumor tissues, we performed immunohistochemical (IHC) analysis of EpCAM staining on a cohort of 153 endometrial carcinoma patients. In total, 98% (150/153) of samples analyzed stained positive for EpCAM (Table S2). Of the positive patients, 80% (*n* = 123) were classified as “EpCAM high”, and 20 % (*n* = 30) as “EpCAM low”. Only 2% (*n* = 3) of patients did not stain for EpCAM. No significant association was found between EpCAM expression and age, International Federation of Gynaecology and Obstetrics (FIGO) stage, histological grade, lymph node metastasis or myometrial infiltration (Table S2). EpCAM was however significantly associated with histologic type, and high EpCAM expression was observed in 84% of patients with endometrioid endometrial carcinoma compared to 64% and 56% of patients with serous endometrial carcinoma and carcinosarcomas, respectively (*p* = 0.002, Table S2). Although 36% of tumors with serous histology were defined as “EpCAM low” according to our cut-off, they demonstrated consistent positive staining (staining index (SI): 3–4 in all cases). No significant association between EpCAM expression and disease specific survival was found in a univariate survival analysis (*p* = 0.49, Figure 1D). To confirm that EpCAM expression is maintained following in vivo passage in immunodeficient mice we isolated cells from a representative PDX model of endometrial carcinoma (PDX2) and performed flow cytometry with an anti-EpCAM antibody. Positive expression of EpCAM was observed (Figure 1E). Together, the data suggest that EpCAM can be targeted in both in vitro and in vivo applications.

### 2.3. EpCAM-AF680 Enables Early Imaging of Metastasis in Cell Line-Based Xenograft Models of Endometrial Carcinoma

**Figure 2:**
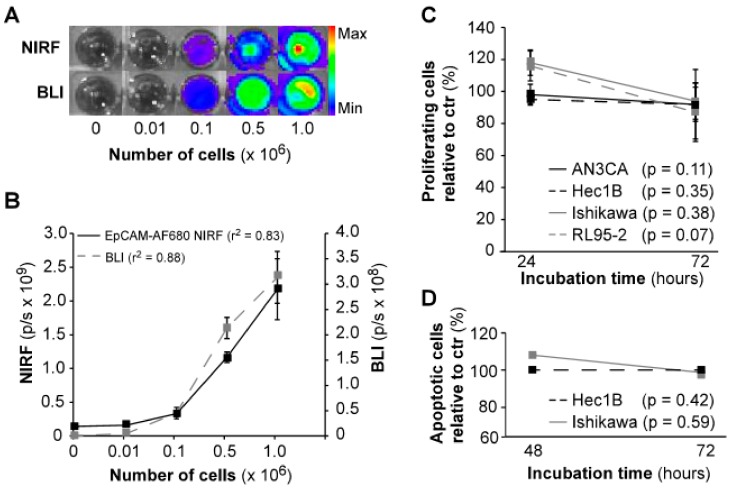
In vitro evaluation of EpCAM-AF680 as a NIRF imaging probe. In vitro imaging of Ishikawa^luc+ ^cells using BLI and EpCAM-AF680 NIRF demonstrates comparable photonic linearity (**A**) and increase in signal (**B**) with higher number of cells (range: 0–10^6^ cells). The lack of significant effects of in vitro incubation with EpCAM-AF680 antibody on proliferation and apoptosis demonstrated by MTS assay (**C**) and Annexin V/PI staining (**D**) in various cell lines. Abbreviations: Bioluminescent imaging (BLI), Epithelial cell adhesion molecule (EpCAM), Near-infrared fluorescent (NIRF), and Propium iodide (PI).

To validate that our EpCAM-AF680 conjugate was able to bind to endometrial carcinoma cells, in vitro, NIRF imaging of luciferase-expressing (luc+) Ishikawa^luc+^ cells was performed. Cells were imaged in parallel using BLI for comparison. Both modalities demonstrated similar increase in signal with higher number of cells, and comparable r^2^ values (BLI: r^2^ = 0.88, EpCAM-AF680: r^2^ = 0.83, Figure 2A,B). In vitro incubation with the EpCAM-AF680 antibody over 72 h did not significantly affect proliferation or apoptosis in any of the cell lines examined (Figure 2C,D).

**Figure 3:**
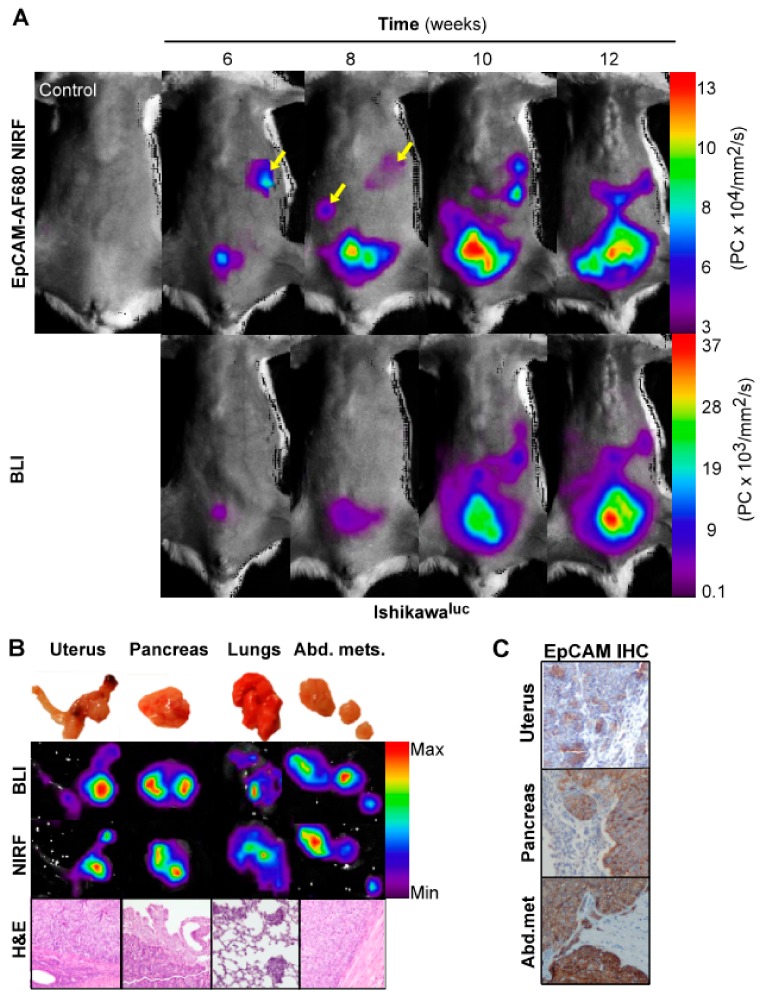
Optical imaging of tumor growth in an orthotopic Ishikawa^luc+^ xenograft model. In vivo BLI and EpCAM-AF680 NIRF imaging of primary tumor growth in mice orthotopically implanted with Ishikawa^luc+^ cells. Metastatic lesions (arrows) were detected at an earlier time point in EpCAM-AF680 NIRF images. A tumor free mouse was used as control (upper left) (**A**). Macroscopic images of uterus, pancreas, lungs and abdominal metastases (**B**, upper panel) and corresponding ex vivo BLI and EpCAM-AF680 NIRF images (**B**, middle panels). Tumor cells are demonstrated in H&E stained sections (20x magnification) (**B**, lower panel). Positive EpCAM expression in primary tumor and metastases is demonstrated by IHC (**C**). Abbreviations: Bioluminescent imaging (BLI), Epithelial cell adhesion molecule (EpCAM), Hematoxylin and eosin (H&E), Immunohistochemistry (IHC), and Near-infrared fluorescence (NIRF).

To validate EpCAM as an imaging biomarker prior to application in PDX models, we wanted to confirm a correlation with NIRF and BLI, which is commonly used for preclinical imaging. Ishikawa^luc+^ cells with high EpCAM expression were therefore orthotopically implanted in mice (*n* = 4), and parallel BLI and EpCAM-AF680 NIRF imaging performed every second week from week 6. Both modalities were able to detect uterine tumors and disease progression from week 6 (Figure 3A). Interestingly, suspected metastases were clearly evident using EpCAM-AF680 from week 6, which were not evident in BLI until week 10 (Figure 3A). After necropsy, the intensity of signal in harvested organs was evaluated by ex vivo BLI and EpCAM-AF680 NIRF imaging. Comparable ex vivo images were generated from the two modalities, and presence of tumor cells in suspected sites of metastasis confirmed by histological evaluation of HE-stained tissue (Figure 3B). Positive EpCAM staining was demonstrated in both uterine tumors and metastases by IHC (Figure 3C).

**Figure 4:**
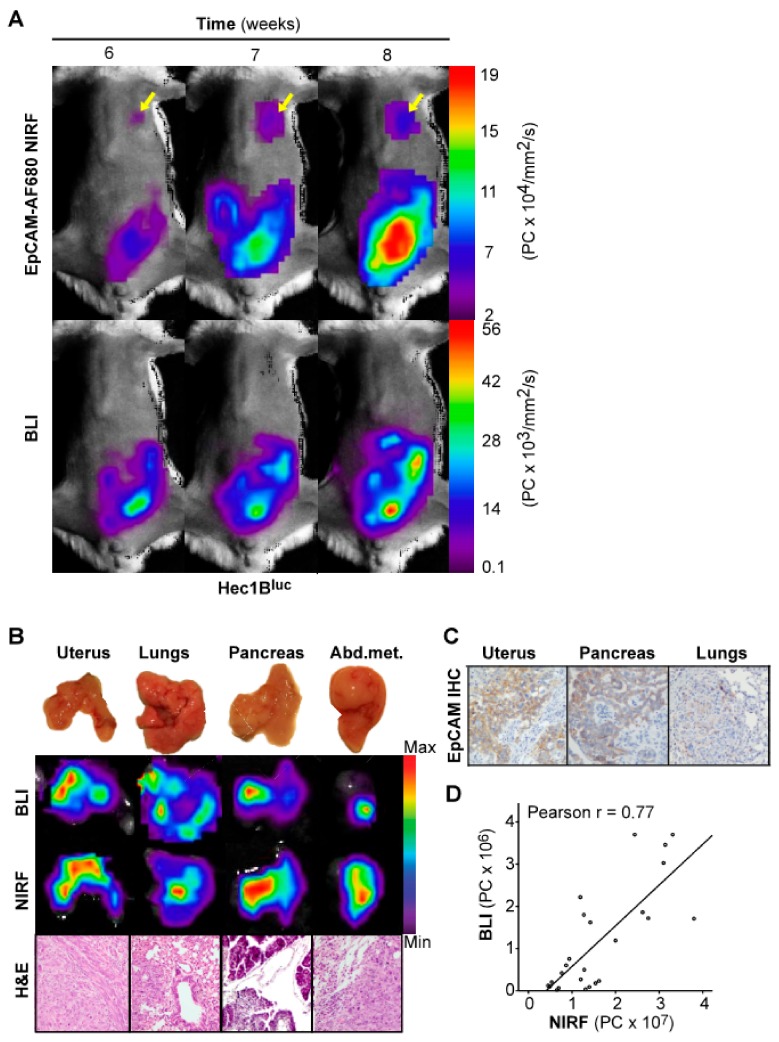
Optical imaging of tumor growth in an orthotopic Hec1B^luc+^ xenograft model. In vivo BLI and EpCAM-AF680 NIRF imaging of primary tumor growth in mice orthotopically implanted with Hec1B^luc+^ cells. NIRF imaging enabled detection of metastatic lesions in the lung (arrows), which were not evident on BLI in vivo (**A**). Macroscopic images of organs harvested during necropsy (**B**, upper panel) and corresponding ex vivo BLI and EpCAM-AF680 NIRF images (**B**, middle panels). Tumor cells are demonstrated in H&E stained sections (20x magnification) (**B**, lower panel). Positive EpCAM expression in uterine tumor and metastases is demonstrated by IHC (**C**). Correlation plot of in vivo NIRF and bioluminescent signal in all mice included in the cell line-based models (**D**). Abbreviations: Bioluminescent imaging (BLI), Epithelial cell adhesion molecule (EpCAM), Hematoxylin and eosin (H&E), Immunohistochemistry (IHC), and Near-infrared fluorescence (NIRF).

To validate that the correlation between EpCAM-AF680 NIRF imaging and BLI was not specific to the Ishikawa^luc+ ^model, an orthotopic xenograft model was also developed from the Hec1B^luc+^ cell line (*n* = 4) and imaged weekly. In this model, both EpCAM-AF680 NIRF and BLI demonstrated comparable capacities in delineating primary tumors (Figure 4A). However, as observed with EpCAM-AF680 NIRF imaging of the Ishikawa^luc+ ^model, distant metastatic lesions were already evident 6 weeks post implantation with the NIRF approach although never detected with corresponding BLI (Figure 4A). Ex vivo BLI and EpCAM-AF680 NIRF imaging correlated well across all infiltrated organs and were confirmed by histology (Figure 4B, Figure S1). Positive EpCAM expression was demonstrated by IHC in both primary tumor and metastatic lesions (Figure 4C). Additionally, we demonstrated a strong correlation between BLI and EpCAM-AF680 NIRF (r = 0.77, Figure 4D).

### 2.4. In Vivo EpCAM-AF680 NIRF Imaging Detects Uterine Tumors in PDX Models of Endometrial Carcinoma

**Figure 5:**
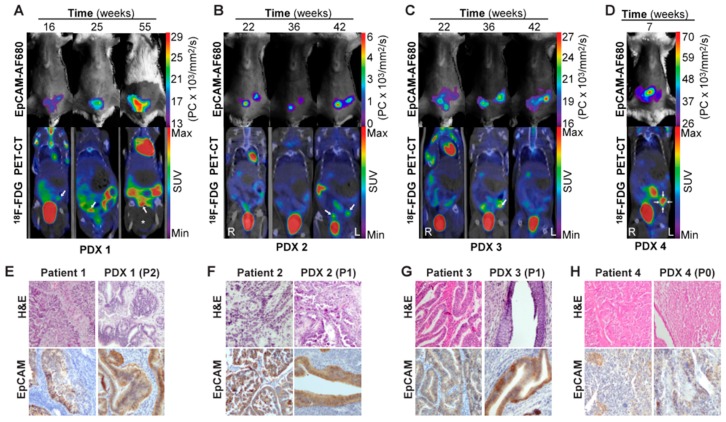
In vivo imaging of tumor growth in PDX models using EpCAM-AF680 NIRF and ^18^F-FDG PET/CT. Longitudinal monitoring of uterine tumors of different histologic types in PDX models using EpCAM-AF680 NIRF and ^18^F-FDG PET/CT imaging. Arrows mark probable uterine tumors in PET/CT images (**A**–**D**). H&E staining demonstrating uterine tumor cells, and positive EpCAM IHC staining of uterine tumors from both donor patients and mouse xenografts (20× magnification) (**E**–**H**). Large bladder removed from image for visualization purposes. An uncropped version of this image can be found in Figure S2. Abbreviations: Epithelial cell adhesion molecule (EpCAM), Fluorine-18-fluorodeoxyglucose (^18^FDG), Hematoxylin and eosin (H&E), Near-infrared fluorescence (NIRF), Patient-derived xenograft (PDX), Positron emission tomography/computed tomography (PET/CT), and Standardized uptake value (SUV).

Having demonstrated EpCAM-AF680 to be useful for preclinical imaging of cell line-based orthotopic xenograft models of endometrial carcinoma, the goal was to apply this imaging strategy to monitor tumor development in PDX models. Primary tumor cells were harvested from endometrial carcinoma patients during hysterectomy and used to generate orthotopic PDX models (Table S3). In vivo EpCAM-AF680 NIRF imaging and fluorine-18-fluorodeoxyglucose (^18^F-FDG) PET/CT imaging were performed in parallel to monitor tumor growth in four different PDX models (Figure 5A–D). Variations in imaging time points of PDX1–4 are caused by different engraftment times for these four models (range: 7–55 weeks). EpCAM-AF680 NIRF imaging enabled visualization of uterine tumors in all four xenografts, demonstrating excellent contrast and strong fluorescent signal (Figure 5A–D). Interestingly, PDX4 developed a uterine tumor that was clearly depicted on EpCAM-AF680 NIRF images (Figure 5D), despite the primary tumor sample for that PDX exhibiting low expression of EpCAM on IHC (SI: 4). PDX mice had suspected uterine tumors based on visual inspection of reconstructed ^18^F-FDG PET/CT images (Figure 5A–D, Figure S2). However, in several of the scans from PDX1–3, suspected lesions had standardized uptake values (SUV) below 2.5, which was the threshold used to define tumors in PET images (Figure 5A–C, Figure S2). Overall, EpCAM-AF680 NIRF imaging appeared to detect tumors at an earlier time point than ^18^F-FDG PET/CT. Uterine tumors were also clearly discerned in EpCAM-AF680 NIRF images, whereas tumors in ^18^F-FDG PET/CT images were more diffuse and difficult to distinguish from normal physiological tracer uptake in the intestine (Figure 5A–D). Histological appearance and EpCAM expression were found to be similar in primary tumor and corresponding xenograft in all cases (Figure 5E–H).

### 2.5. EpCAM-AF680 NIRF Imaging of Therapeutic Efficacy in An Orthotopic Endometrial Carcinoma PDX Model

**Figure 6:**
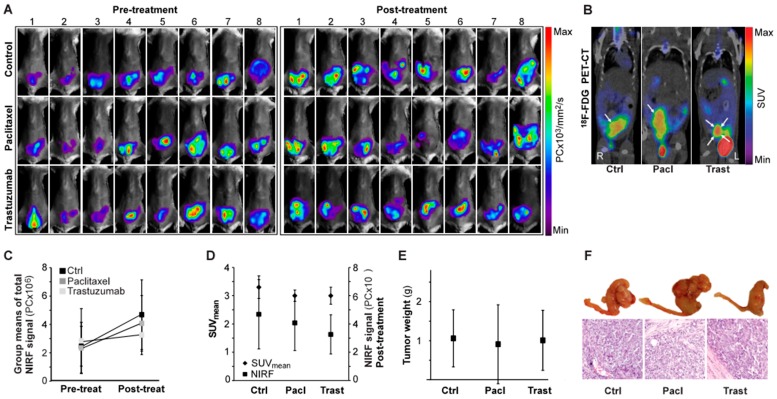
In vivo monitoring of paclitaxel or trastuzumab treatment in an endometrial carcinoma PDX model using EpCAM-AF680 NIRF imaging. EpCAM-AF680 NIRF images of mice treated with control vehicle (*n* = 8), paclitaxel (*n* = 8) or trastuzumab (*n* = 8) prior to (day 26) and after (day 55) treatment. Pre-and post-treatment scans were paired and signal intensity scale synchronized for each individual mouse (**A**). Representative ^18^F-FDG PET/CT images (day 47), uterine tumors are indicated by arrows (**B**). Group means of total fluorescent signal before (day 26) and after (day 55) treatment, *n* = 8 mice per group (**C**). Group means of total fluorescent signal after treatment (day 55, *n* = 8 mice per group) compared to group SUV_mean_ values measured in ^18^F-FDG PET/CT scans at day 47, control group: *n* = 6, paclitaxel group: *n* = 5, trastuzumab: *n* = 6 (**D**). Tumor weights after necropsy on day 49 (control group: mouse 1, paclitaxel group: mouse 2), day 51(control group: mice 3 and 8, trastuzumab group: mouse 2) or day 57 (all other mice), *n* = 8 in all groups (20× magnification) (**E**,**F**). Graphs are group means +/− SD. Abbreviations: Epithelial cell adherence molecule (EpCAM), Fluorine-18-fluorodeoxyglucose (^18^F-FDG), Metabolic tumor volume (MTV), Near infrared fluorescence (NIRF), Positron emission tomography/computed tomography (PET/CT), and Standardized uptake value (SUV).

Having established that NIRF imaging with EpCAM-AF680 could be employed to non-invasively monitor tumor growth in PDX models in a spatio-temporal manner, a cohort of mice (*n* = 24) were orthotopically implanted with cells from a patient diagnosed with grade 3 endometrioid endometrial carcinoma (PDX4) and treated with paclitaxel (*n* = 8), trastuzumab (*n* = 8) or vehicle control (*n* = 8). Each mouse underwent EpCAM-AF680 NIRF imaging prior to initiation of therapy and post-treatment (Figure 6A). Mice were also imaged once using ^18^F-FDG PET/CT (Figure 6B). Total fluorescence was measured to evaluate the change in disease status over time (Figure 6C). Although not significant (F(2,21) = 1.04, *p* = 0.37), there was a tendency toward lower NIRF signal in the paclitaxel (mean: 4.1 × 10^6^ +/− 2.0 × 10^6^ PC) and trastuzumab (mean: 3.3 × 10^6^ +/− 1.4 × 10^6^ PC) treated groups compared to controls (mean: 4.7 × 10^6^ +/− 2.5 × 10^6^ PC) post treatment (Figure 6C). While xenografts could be clearly discerned in all mice using EpCAM-AF680 NIRF, for six mice no uterine tumor was identified on ^18^F-FDG PET/CT (not shown). One mouse had missing PET data due to technical problems. Mean standardized uptake values (SUV_mean_) of uterine tumors were estimated (group mean values: control group; 3.3 +/− 0.4, paclitaxel group; 3.0 +/− 0.2, trastuzumab group; 3.0 +/− 0.3 mm^3^), but no significant differences were observed between groups (F(2,14) = 1.37, *p* = 0.29, Figure 6D). Following necropsy tumors were weighed. Consistent with findings from EpCAM-AF680 NIRF imaging there were no statistically significant differences in tumor weights between groups (mean weight control group; 1.06 +/− 0.73 g, paclitaxel group; 0.91 +/− 1.01 g, trastuzumab group; 1.01 +/− 0.77 g) (F(2,21) = 0.07, *p* = 0.93, Figure 6E). Histological examination of uterine tissue confirmed presence of tumor cells in all samples (Figure 6F). 

## 3. Discussion

Amongst the major challenges in endometrial carcinoma is the lack of treatment alternatives targeting specific molecular alterations. PDX models are considered clinically relevant for studies of targeted therapies, but monitoring of disease development, staging and treatment response is challenging in an orthotopic setting. Non-invasive imaging modalities are auspicious tools to overcome this problem as we have previously reported for BLI, MRI and PET/CT (with both ^18^F-FDG and ^18^F-fluorothymidine as tracers) in cell line xenograft studies [18]. However, extension of these modalities to the visualization of orthotopic PDX models required a more targeted approach. Previously, NIRF imaging using EpCAM antibodies have been demonstrated to detect orthotopic tumors in cell line-based xenograft models of head and neck, breast and colorectal cancer [24], as well as to visualize metastatic lymph nodes [34] and improve detection of tumor margins [35] in preclinical prostate cancer models. In the current study, we extend this approach to develop a comprehensive NIRF imaging probe EpCAM-AF680 and demonstrate its feasibility in monitoring of orthotopic endometrial carcinoma PDX models.

There are several benefits of using antibody-based NIRF imaging compared to other imaging modalities. BLI is commonly used for preclinical imaging, but relies on genomic introduction of reporter genes [36]. Optical imaging of EpCAM-AF680 is also directly applicable to new endometrial carcinoma PDX models, whereas transfection of reporter genes must be repeated each time a new model is generated. In the present study, we find that EpCAM-AF680 NIRF enables identification of metastases. This is a major strength, as correct evaluation of disseminated disease is important for both preclinical purposes as well as clinical management of disease [37]. Further, with NIRF imaging there is no need for radioactive tracers. This sets fewer demands to facilities and staff, reduces costs and makes NIRF imaging a more available modality for preclinical imaging. Additionally, application of NIRF imaging adheres to the principle of the 3R’s as scan time is shorter compared to PET/CT (refinement), and as the use of orthotopic PDX models yields clinically relevant information that may limit the number of animals needed (reduction) [38].

To demonstrate the capacity of EpCAM-AF680 NIRF imaging to monitor tumor development in a therapeutic setting, we imaged mice (PDX4) before and after treatment with paclitaxel or trastuzumab. Paclitaxel is a part of the first-line adjuvant chemotherapeutic treatment of endometrial carcinoma [39,40], while trastuzumab is an antibody targeting human epidermal growth factor 2 (HER2) that has been used for selected patients with endometrial carcinoma [41]. Patient 4 (from whom PDX4 was generated) was treated with paclitaxel/carboplatin treatment but suffered disease recurrence 8 months after primary surgery and later died from her disease. As the patient had positive HER2 expression in tumor, we also wanted to treat PDX4 with trastuzumab to determine whether this could improve the outcome. Unfortunately, no statistically significant effects of trastuzumab treatment were detected, either by EpCAM-AF680 NIRF imaging or necropsy findings. Even though none of these drugs were found to inhibit tumor development, the ability of EpCAM-AF680 NIRF imaging to capture therapeutic response in vivo shows promise for use in future treatment studies in endometrial carcinoma PDX models. However, as our results are based on treatment of a single PDX model, the imaging method should be validated in multiple PDX models—preferably representing all subtypes of the disease. It will also be important to validate the clinical relevance of new PDX models to ensure that therapeutic response corresponds to that of their human donors. EpCAM-AF680 NIRF imaging should also be used to monitor multiple treatment experiments with drugs exhibiting different degrees of therapeutic response, in order to validate the clinical accuracy of the method as well as to establish a cut-off for the lower detection limit.

Although NIRF is a powerful tool for imaging of cancer, there are some limitations. NIRF imaging is not quantitative, and the lack of three-dimensional information makes it difficult to precisely identify the exact anatomical location of tumors [36]. Some of these challenges may be overcome by linking EpCAM-antibodies to radioactive isotopes and use them as PET tracers. This has been done with the bispecific EpCAM/CD3ε binding antibody AMG 110, which has been linked to ^89^Zr and used to detect tumors in colorectal and head and neck cancer xenografts [42]. Combining the tumor-targeting properties of EpCAM with three-dimensional PET/CT technology may create a translational imaging tool that improves the value of both preclinical and diagnostic imaging of endometrial carcinoma.

EpCAM may also serve as an imaging target for image-guided surgery (IGS). Sentinel lymph node removal is increasingly applied in endometrial carcinoma [43,44], but the fluorophore commonly used for IGS (indocyanine green) is not tumor-specific [45,46]. Development of tumor-targeting probes may improve the clinical utility. In our present study EpCAM-AF680 NIRF imaging allowed early detection of metastases in endometrial carcinoma mouse models, indicating that EpCAM is expressed in metastatic lesions. This, combined with the observed high proportion of endometrial tumors with positive EpCAM staining in our cohort, suggests EpCAM as a potential biomarker for IGS that should be further explored in future studies.

## 4. Materials and Methods

### 4.1. Monoclonal Antibodies for In Vitro Studies

To identify potential target molecules for NIRF imaging, databases (www.proteinatlas.org and www.uniprot.org) were searched for proteins located on the surface of endometrial carcinoma cells. Candidate proteins with reported overexpression in endometrial carcinoma compared to normal epithelium (ALCAM, EpCAM and IGF1Rα) or with positive expression associated with aggressive disease (L1CAM) were selected [26,28,29,30,31,32,33]. Direct PE-conjugated monoclonal antibodies (Mouse Anti-EpCAM PE (clone EBA-1), PE Mouse Anti-Human CD221 (clone 1H7), PE Mouse Anti-Human CD166 (clone 3A6) (all BD Biosciences, San Jose, CA, USA) and Mouse Anti-Human CD171 Antibody (PE) (clone 03, Sino Biological Inc., Beijing, China) were purchased and tested by flow cytometry to evaluate target expression in cell lines.

### 4.2. Cell Lines

The endometrial adenocarcinoma cell lines AN3CA, Hec1B, RL95–2 (all American Type Culture Collection; Manassas, VA, USA), and Ishikawa (Sigma-Aldrich, St. Louis, MO, USA) were cultured in a humidified atmosphere with 5% CO_2_, at 37 °C, under conditions as recommended by the suppliers. All cell line growth mediums were supplemented with 2 mM L-glutamine and 100 IU/mL penicillin + 100 µg/mL streptomycin (all; Lonza, Basel, Switzerland).

### 4.3. Flow Cytometry

In total, 0.5 × 10^6^ cells (cell lines) or 0.2 × 10^6 ^cells (patient cells) per sample were washed twice and resuspended in 50 µL wash buffer (1% bovine serum albumin (BSA)/phosphate buffered saline (PBS)). Samples were protected from light while incubated for 30 min on ice with PE-conjugated antibodies. After incubation cells were washed twice with 1% BSA/PBS and resuspended in 150 µL PBS. Samples were analyzed using an Accuri™ C6 (BD Biosciences, San Jose, CA, USA) flow cytometry system, and data processed using CFlow Sampler Analysis 1.0.227.4 software.

### 4.4. Validation of EpCAM as Relevant Marker for Endometrial Carcinoma

A cohort of 153 endometrial carcinoma patients representative of the Bergen Gynecologic Biobank was selected, and used for evaluation of EpCAM protein expression. Clinical and histopathological information was retrieved from medical records. Formalin fixed paraffin embedded tissue from primary endometrial carcinomas was used to generate tissue microarrays as previously described [47]. IHC staining was performed according to a standardized institutional protocol [18]. EpCAM was detected using a monoclonal antibody (D9S3P; Cell Signaling Technologies, Danvers, MA, USA [48]) under the following conditions: pH 6, 1:200 dilution, incubated for 60 min at room temperature. SI was calculated for each patient as previously described [47]. Briefly, slides are given one score of 0–3 based on staining intensity (0 = no staining, 3 = strong staining) and one score of 0–3 based on the area of the tumor with positive staining (0 = no staining, 1 = less than 10%, 2 = 10%–50%, 3 = >50%). The two scores are then multiplied, giving a final SI within the range of 0–9. Both cytoplasmic and membranous staining of EpCAM was observed in tumor cells, and overall staining was evaluated without considering sub-cellular localization. “EpCAM high” was defined as SI: 6–9, while “EpCAM low” was defined as SI: 0–4.

### 4.5. Alexa Fluor 680 (AF680) Conjugation of EpCAM Antibody

A monoclonal mouse anti-human low endotoxin anti-EpCAM antibody (MCA1870EL, clone VU-1D9; BioRad, Hercules, CA, USA) was purchased for in vivo experiments. Anti-EpCAM antibody was conjugated to AF680 using the SAIVI Rapid Antibodies Labeling Kit (Invitrogen/Thermo Fisher Scientific Inc., Waltham, MA, USA) following instructions from the manufacturer. A Nanodrop 1000 spectrophotometer (Thermo Scientific, Waltham, MA, USA) was used to determine protein concentration and degree of labeling. To confirm fluorescent signal, series of cells (0, 10^4^, 10^5^ and 10^6^) were seeded in 96-well plates as 100 µL cell suspensions and incubated with 0.4 µg EpCAM-AF680 for 60 min on ice while protected from light. Excess antibody was removed by washing with 1% BSA/PBS. In vitro NIRF imaging and data analyses were performed using the IVIS Spectrum In Vivo Imaging System (PerkinElmer, Waltham, MA, USA).

### 4.6. Cell Transfection, Viability and Proliferation Assays

Ishikawa and Hec1B cells were stably transfected with a luciferase-expressing construct by retroviral infection, as previously described [18]. Luc^+^ cells (Hec1B^luc+^, Ishikawa^luc+^) were selected using 1 µg/mL puromycin (Sigma-Aldrich, St. Louis, MO, USA). Luciferase activity was confirmed by seeding series of cells (0, 10^4^, 10^5^, and 10^6^) in 96-well plates as 100 µL cell suspensions, and performing in vitro BLI 10 min after addition of d-luciferin (2.5 mg/mL; Promega, Madison, WI, USA) using the IVIS Spectrum In Vivo Imaging System (PerkinElmer, Waltham, MA, USA).

Proliferation was assessed using the CellTiter 96® AQueous One Solution Cell Proliferation Assay (Promega, Madison, WI, USA) as described by the supplier. AN3CA, Hec1B^luc+^, Ishikawa^luc+^, and RL95–2 cells were seeded in 96-well plates and incubated with EpCAM-AF680 antibody. Absorbance (490 nm) was recorded one hour after adding 20 µL of substrate, using a TECAN Magellan Sunrise plate reader and TECAN Magellan software version 6.3 (both Tecan, Männedorf, Switzerland). All experiments were performed in triplicates.

Evaluation of apoptosis was performed by Annexin V/propium iodine (PI) staining. Cells were incubated with EpCAM-antibody (MCA1870EL), harvested, washed twice in PBS, and suspended in 1x binding buffer (Thermo Fisher Scientific, Waltham, MA, USA). APC Annexin V and PI (both Thermo Fisher Scientific, Waltham, MA, USA) were applied for 15 min at room temperature protected from light, and samples immediately analyzed by flow cytometry.

### 4.7. Orthotopic Mouse Models

Female NOD/SCID IL2rγ^null^ (NSG) mice were bred at the University of Bergen animal facilities or purchased from Charles River Laboratories (Saint-Germain-Nuelles, France), and kept in individually ventilated cages with a maximum of 5 mice in each cage. Ad libitum access to food and water was provided. Mice were fed with low-autofluorescence rodent imaging diet (2018S Teklad Global 18% Protein Rodent Diet, Envigo, Cambridgeshire, UK, or D10001, Research Diets Inc., New Brunswick, NJ, USA) for at least two weeks prior to imaging. Endometrial carcinoma cells were implanted orthotopically in the left uterine horn as previously reported [18]. Mice were monitored for lethargy, ataxia and abdominal enlargement, and were sacrificed by cervical dislocation following any of these symptoms or weight loss of ≥10%.

Cell line-based orthotopic models were generated by uterine implantation of 1 × 10^6^ Hec1B^luc+^ (*n* = 4) or Ishikawa^luc+^ (*n* = 4) cells. Parallel BLI and EpCAM-AF680 NIRF imaging were performed either weekly (Hec1B^luc+^) or every other week (Ishikawa^luc+^), starting 6 weeks after implantation. *Ex vivo* imaging of organs was carried out immediately after euthanasia.

Biopsies from human primary tumors were collected during hysterectomy and kept on ice until processing. Tumor tissue was prepared by manual dissociation as previously described [18], and cells were suspended in matrigel prior to orthotopic implantation in mice (P0-generation). When mice were in a moribund state, PDX models were maintained by re-implantation in new mice (P1-generation, etc.). In total, PDX models generated from 4 patients have been included in this study. Tumor growth in PDX mice was monitored in parallel using EpCAM-AF680 NIRF- and dynamic ^18^F-FDG PET/CT imaging.

### 4.8. EpCAM-AF680 NIRF Imaging for Monitoring of Therapeutic Effect in An Endometrial Carcinoma PDX Model

Primary human tumor cells (PDX4) were isolated from hysterectomy specimen, expanded in short-time culture and orthotopically implanted in 24 mice (1 × 10^6^ cells per mouse). Then, 28 days after implantation, animals were randomized into three treatment groups (*n* = 8 mice per group). Baseline NIRF signal, body weight and time of implantation were evaluated by ANOVA-testing to ensure that there were no significant differences between groups prior to starting treatment. Mice were treated with 10 mg/kg trastuzumab i.p. once weekly [49] or 12 mg/kg paclitaxel i.p. twice weekly [50]. The control group received equivalent volumes of saline twice weekly. As trastuzumab was administered only once weekly, mice in the trastuzumab group were injected with saline once a week in order to receive the same number of injections as mice in the other treatment groups. EpCAM-AF680 NIRF imaging was performed prior to starting treatment (day 26) and after treatment (day 55). ^18^F-FDG PET/CT imaging was performed on day 47. Mice were fasted 12 h prior to PET/CT imaging to reduce tracer uptake in the gut. Animals were sacrificed when developing signs of clinical disease, or at day 57 (final endpoint). After necropsy, tumors were weighed, and tissue samples collected for histological evaluation. The main events of the treatment study are presented in Figure S3.

### 4.9. In Vivo BLI

Mice implanted with Luc^+^ cells were injected with 150 mg/kg d-luciferin (Promega, Madison, WI, USA) i.p. 10 min prior to imaging. Animals were anesthetized using 3% isoflurane (IsoFlo vet. Zoetis, Parsippany, NJ, USA), + 1 L/min O_2_ for induction, and maintained with 1.5–2% isoflurane + 1.0 L/min O_2_ for imaging. Bioluminescent images were obtained using an Optix MX3 Time-Domain Optical Imager (ART Inc., Saint-Laurent, QC, Canada) with raster scan points 1.5 mm apart. Images were analyzed using the Optix OptiView software (version 2.02; ART Inc., Saint-Laurent, QC, Canada). A region of interest (ROI) was manually drawn around the abdomen and thorax to measure total bioluminescent signal in in vivo scans. For ex vivo images, ROIs were manually drawn around each organ.

### 4.10. In Vivo NIRF

Then, 24 h prior to imaging, animals were injected with the EpCAM-AF680 antibody in the tail vein (cell line models: 50 µg/mouse, PDX1–3: 75 µg/mouse, and PDX4 + treatment study: 60 µg/mouse). Animals were depilated before imaging, and urine was manually removed to avoid excess fluorescent signal from the bladder. EpCAM-AF680 NIRF images were obtained using the Optix MX3 Time-Domain Optical Imager (ART Inc., Saint-Laurent, QC, Canada), λ_ex_ = 670 nm, λ_em_ = 700 LP, raster scan points 1.5 mm apart. Image analysis was performed using the Optix OptiView software (version 2.02; ART Inc., Saint-Laurent, QC, Canada). A tumor free mouse injected with EpCAM-AF680 was imaged in parallel as control. In cell line models a ROI was manually drawn around the abdomen and thorax to measure total fluorescent signal of in vivo scans. For ex vivo images, ROIs were manually drawn around each organ. In the PDX treatment study, background signal was removed prior to measuring the fluorescent signal. For visualization of tumor progression, pre- and post-treatment images were paired, and the signal intensity scale was synchronized for each mouse.

### 4.11. F-FDG PET/CT Acquisition and Image Analysis

Integrated PET/CT whole-body images were collected using a nanoScan PET/CT (Mediso Medical Imaging Systems Ltd, Budapest, Hungary), with spatial resolution 800 µm (PET) and 30 µm (CT) as previously described [18]. Animals were anesthetized with 3% sevoflurane (SevoFlo vet., Zoetis, Parsippany, NJ, USA) during tracer injection and imaging. A CT uptake (50 kVp, 0.1 mAs) was performed for attenuation correction and anatomical localization of the PET signal. ^18^F-FDG was injected i.v. in the tail vein at start of the 1 h PET acquisition, and static attenuation corrected PET images with voxel size of 0.4 mm^3^ were reconstructed summing the last 30 min of the scan. PET/CT images were reviewed for tumor uptake and/or other pathologically suspicious areas. Measurements of SUV_mean_ included voxels with SUV above 2.5, as previously reported for endometrial carcinoma patients [51].

### 4.12. Statistical Analyses

SPSS Statistics software (version 24.0; IBM Corp., Armonk, NY, USA) was used for statistical analyses. All statistical tests were two-sided, and *p*-values ≤ 0.05 were regarded statistically significant. Disease-specific survival curves were generated using the Kaplan–Meier method, and survival between groups compared using the log rank (Mantel–Cox) test. In the patient series time of surgery was used as entry date, and time to death due to endometrial carcinoma was defined as endpoint. Categorical variables were evaluated using the Pearson Chi-square test. Linear correlation between continuous variables was evaluated by calculating the Pearson correlation coefficient. One-way analysis of variance was performed to compare group means in the PDX treatment study.

### 4.13. Approvals

All animal experiments were approved by the Norwegian State Commission for Laboratory Animals (FOTS ID 6735) and have been conducted according to the European Convention for the Protection of Vertebrates Used for Scientific Purposes. Ethical approval for inclusion of patients has been granted by the Western Regional Committee for Medical and Health Research Ethics (REK 2009/2315, REK 2014/1907, REK 2018/594). Written informed consent was obtained from all participating patients prior to inclusion in the Bergen Gynecologic Biobank.

## 5. Conclusions

EpCAM-AF680 NIRF imaging enables evaluation of treatment response in clinically relevant endometrial carcinoma PDX models and may facilitate translational studies of new targeted treatments and corresponding response predicting markers. EpCAM may serve as a translational imaging biomarker and is a potential target for IGS.

## Supplementary Materials

The following are available online at https://www.mdpi.com/2072-6694/12/2/370/s1. Figure S1: Lung metastases in Hec1Bluc+ cell line model; Figure S2: Timeline for treatment study; Figure S3: Timeline demonstrating important events in the PDX model paclitaxel/trastuzumab treatment study; Table S1: Fold increase in in vitro MFI in PE-stained endometrial carcinoma cell lines; Table S2: EpCAM expression related to clinicopathological variables in 153 endometrial carcinoma patients; Table S3. Characteristics of patient-derived xenografts.
