# Supplementary material for: Near-Infrared Fluorescent Imaging for Monitoring of Treatment Response in Endometrial Carcinoma Patient-Derived Xenograft Models
Source: Cancers (Basel). 2020 Feb 6;12(2):370. doi: 10.3390/cancers12020370 (PMC7072497; doi:10.3390/cancers12020370)
Supplement: Supplementary file 1 [file cancers-12-00370-s001.zip › Figure S1.pdf]

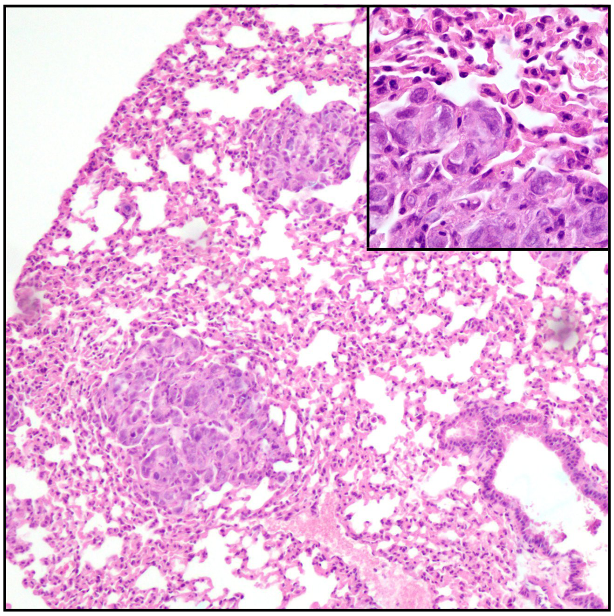

Figure S1. Lung metastases in Hec1Bluc+ cell line model
Presence of lung metastases were confirmed by histology in a mouse orthotopically implanted with Hec1Bluc+ cells. The margin of a metastatic lesion is magnified (x 4 compared to main image) in the upper right corner (insert).
