# Supplementary material for: Near-Infrared Fluorescent Imaging for Monitoring of Treatment Response in Endometrial Carcinoma Patient-Derived Xenograft Models
Source: Cancers (Basel). 2020 Feb 6;12(2):370. doi: 10.3390/cancers12020370 (PMC7072497; doi:10.3390/cancers12020370)
Supplement: Supplementary file 1 [file cancers-12-00370-s001.zip › Figure S3.pdf]

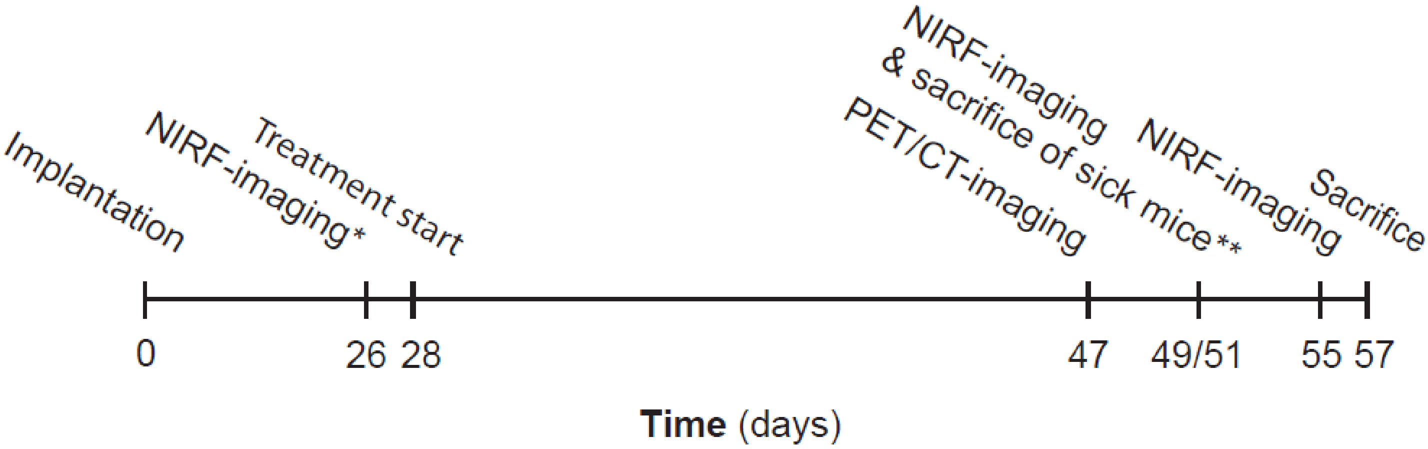

Figure S3. Timeline demonstrating important events in the PDX model paclitaxel/trastuzumab treatment study.

*After mice were randomized into treatment groups

**5 mice were euthanized due to clinical signs of disease, and underwent NIRF imaging immediately prior to sacrifice.

Abbreviations: Near infrared fluorescence (NIRF), Patient-derived xenograft (PDX), Positron emission tomography/computed tomography (PET/CT)
