# Supplementary material for: Near-Infrared Fluorescent Imaging for Monitoring of Treatment Response in Endometrial Carcinoma Patient-Derived Xenograft Models
Source: Cancers (Basel). 2020 Feb 6;12(2):370. doi: 10.3390/cancers12020370 (PMC7072497; doi:10.3390/cancers12020370)
Supplement: Supplementary file 1 [file cancers-12-00370-s001.zip › Table S1.docx]

**Table S1. Fold increase of *in vitro* MFI in PE-stained endometrial carcinoma cell lines.**

| **Cell line:** | **MFI (PE) fold increase^a^** | | | |
| --- | --- | --- | --- | --- |
|  | **ALCAM** | **EpCAM** | **IGF1Rα** | **L1CAM** |
| **AN3CA** | 66.8 (88.9) | 29.8 (48.5) | 10.6 (13.7) | 9.0 (34.8) |
| **Hec1B** | 39.7 (99.2) | 89.1 (99.2) | 12.6 (66.7) | 50.7 (96.2) |
| **Ishikawa** | 56.6 (99.9) | 305.3 (99.9) | 9.8 (4.6) | 12.3 (0.7) |
| **RL-952** | 13.0 (31.2) | 41.2 (96.4) | 11.8 (2.5) | 20.8 (40.5) |

^a^Evaluated by flow cytometry, relative to unstained cells. % of cells with positive staining in brackets

Abbreviations: Mean fluorescent intensity (MFI), phycoerythrin (PE).
