# Supplementary material for: Near-Infrared Fluorescent Imaging for Monitoring of Treatment Response in Endometrial Carcinoma Patient-Derived Xenograft Models
Source: Cancers (Basel). 2020 Feb 6;12(2):370. doi: 10.3390/cancers12020370 (PMC7072497; doi:10.3390/cancers12020370)
Supplement: Supplementary file 1 [file cancers-12-00370-s001.zip › Table S2.docx]

**Table S2. EpCAM expression related to clinicopathological variables in 153 endometrial carcinoma patients**

|  |  | | EpCAM staining index | |  |
| --- | --- | --- | --- | --- | --- |
| Variable | **N** (153) | | **Low *n* (%)^a^** | **High *n* (%)^b^** | **P-value (*χ*^2^)** |
| Age | 153 | |  |  | 0.339 |
| < 66 | 68 | | 11 (16) | 57 (84) |  |
| ≥ 66 | 85 | | 19 (22) | 66 (78) |  |
| FIGO stage 2009 | 153 | |  |  | 0.265 |
| I-II | 132 | | 24 (18) | 108 (82) |  |
| III-IV | 21 | | 6 (29) | 15 (71) |  |
| Histologic type | 153 | |  |  | 0.002 |
| Endometrioid | 121 | | 19 (16) | 102 (84) |  |
| Clear cell | 7 | | 0 (0) | 7 (100) |  |
| Serous | 14 | | 5 (36) | 9 (64) |  |
| Carcinosarcoma | 9 | | 4 (44) | 5 (56) |  |
| Undifferentiated | 2 | | 2 (100) | 0 (0) |  |
| Histologic grade^c^ | 116 | |  |  | 0.543 |
| Grade 1-2 | 96 | | 14 (15) | 82 (85) |  |
| Grade 3 | 20 | | 4 (20) | 16 (80) |  |
| Myometrial infiltration | | 153 |  |  | 0.620 |
| <50% | 96 | | 20 (21) | 76 (79) |  |
| ≥50% | 57 | | 10 (17) | 47 (83) |  |

^a^ In total 30 patients with low EpCAM staining

^b^ In total 123 patients with high EpCAM staining

^c^ Endometrioid cases only. Missing information on histologic grade for 5 patients
