# Supplementary material for: Near-Infrared Fluorescent Imaging for Monitoring of Treatment Response in Endometrial Carcinoma Patient-Derived Xenograft Models
Source: Cancers (Basel). 2020 Feb 6;12(2):370. doi: 10.3390/cancers12020370 (PMC7072497; doi:10.3390/cancers12020370)
Supplement: Supplementary file 1 [file cancers-12-00370-s001.zip › Table S3.docx]

**Table S3. Characteristics of patient derived xenografts**

|  | **PDX1** | **PDX2** | **PDX3** | **PDX4** |
| --- | --- | --- | --- | --- |
| **Age** | 69 | 75 | 65 | 58 |
| **FIGO stage** | IB | IIIC1 | IB | IB |
| **Histologic type** | Endometrioid | Serous | Endometrioid | Endometrioid |
| **Histologic grade** | 3 | 3 | 1 | 3 |
| **Xenograft generation** | P2 | P1 | P1 | P0 |
|  |  |  |  |  |
