# Supplementary figures and images for: Near-Infrared Fluorescent Imaging for Monitoring of Treatment Response in Endometrial Carcinoma Patient-Derived Xenograft Models
Source: Cancers (Basel). 2020 Feb 6;12(2):370. doi: 10.3390/cancers12020370 (PMC7072497; doi:10.3390/cancers12020370)

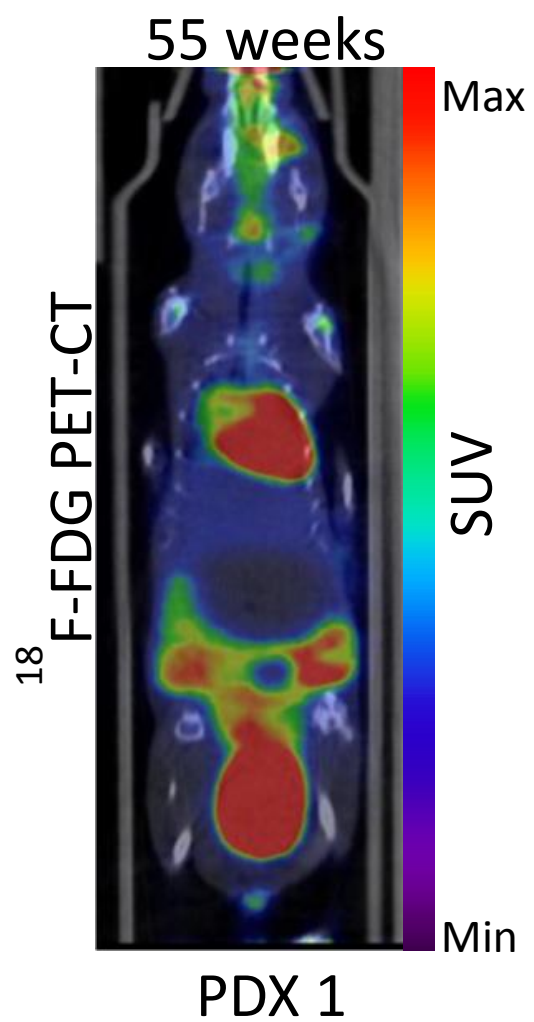

**Figure S2.** Uncropped  $^{18}\text{F}$ -FDG PET-CT image of PDX 1 55 weeks post implantation.

Supplement: Supplementary file 1 [file cancers-12-00370-s001.zip › Figure S2.pdf]
